# Effectiveness of Hysteroscopic Surgery for Intrauterine Lesions on Pregnancy Rates in Patients With Primary Infertility in Bahrain

**DOI:** 10.7759/cureus.92694

**Published:** 2025-09-19

**Authors:** Aalaa A Marzooq, Batool J Marhoon, Huda A Ali

**Affiliations:** 1 Obstetrics and Gynaecology, Salmaniya Medical Complex, Manama, BHR

**Keywords:** hysteroscopy, intrauterine lesions, polyps, pregnancy rates, primary infertility, submucosal fibroids, uterine septa

## Abstract

Background

Infertility poses a substantial challenge in the realm of reproductive health, impacting numerous couples globally. Intrauterine abnormalities like polyps, submucosal fibroids, and uterine septa can impede the process of successful implantation and placentation, thereby contributing to infertility issues and miscarriages. Hysteroscopy has emerged as a promising therapeutic approach for addressing these intrauterine lesions. Our objective is to assess the efficacy of hysteroscopic surgery in treating intrauterine lesions and its impact on pregnancy rates among individuals experiencing primary infertility.

Objectives

Our primary objective is to assess the association between operative hysteroscopy for defined intrauterine lesions (polyps, submucous fibroids, septa) and subsequent pregnancy and live-birth rates in women with primary infertility. Secondary objectives: (i) quantify the proportion of previously undiagnosed lesions detected at hysteroscopy; (ii) explore predictors of pregnancy (age, BMI, infertility duration, and lesion type); and (iii) report outcomes stratified by conception method (spontaneous vs. assisted reproductive technology (ART)).

Material and methods

This retrospective cohort study was conducted in the Obstetrics and Gynaecology department at Salmaniya Medical Complex (SMC), Bahrain. The study included 113 patients with primary infertility due to intrauterine lesions, such as polyps, submucosal fibroids, and uterine septa, who underwent hysteroscopic surgery at SMC between September 2018 and February 2023. The primary outcome of interest was the achievement of pregnancy following hysteroscopic surgery. Secondary outcomes included the mode of conception (spontaneous or assisted). Pregnancy outcomes were confirmed through registry follow-up to ensure accuracy and reproducibility of the data.

Results

After operative hysteroscopy, 72 out of 113 patients (64%) achieved pregnancy. Among those who became pregnant, 73.6% had singleton pregnancies, while 26.4% had twin pregnancies. Of the patients who achieved pregnancy, 18.1% experienced miscarriage, 1.4% had an ectopic pregnancy, 62.2% carried to term, and 18.1% had preterm deliveries. The overall live birth rate was 80.6% (58 live births).

The highest live birth rate was observed in the polypectomy group (37.9%), followed by the uterine septum resection group (31%), the diagnostic hysteroscopy and endometrial scratching group (15.5%), the hysteroscopic myomectomy group (8.6%), and the hysteroscopic polypectomy and myomectomy group (6.9%).

Conclusions

Hysteroscopic surgery for intrauterine lesions significantly improved pregnancy rates in patients with primary infertility, proving particularly effective for treating polyps, uterine septa, and submucosal fibroids. These findings underscore the importance of hysteroscopy as a valuable treatment option for patients with primary infertility and intrauterine abnormalities.

## Introduction

Infertility is a disease of the male or female reproductive system defined by the failure to achieve a pregnancy after 12 months or more of regular unprotected sexual intercourse, affecting millions of couples worldwide [1]. It is widely recognised that infertility can considerably affect an individual's quality of life, with a significant psychological impact. Studies have shown high rates of depression and anxiety symptoms among people with infertility [2]. Psychological distress may also exacerbate infertility, making it a significant medical and social problem.

Gynaecological conditions influencing implantation rates include endometriosis, hydrosalpinges, and intrauterine lesions such as polyps, fibroids, and adhesions [3].

Hysteroscopic surgery is a minimally invasive procedure that allows direct visualisation and removal of intrauterine lesions and is an effective treatment option for women with primary infertility [4]. It has revolutionised the diagnosis and management of intrauterine abnormalities in gynaecological practice and is widely used for both diagnostic and therapeutic purposes [5].

Several studies have investigated the effectiveness of hysteroscopic surgery for intrauterine lesions on pregnancy rates in patients with primary infertility. While some studies have reported conflicting results, the evidence suggests that hysteroscopic surgery can improve pregnancy rates in this population. For instance, a systematic review and meta-analysis conducted by Nouri et al. (2010) found that hysteroscopic surgery for intrauterine lesions was associated with a significant increase in clinical pregnancy rates compared to no treatment or conservative management [6].

However, it is important to note that the success of hysteroscopic surgery may depend on various factors, such as the size and location of the lesion, the presence of other infertility factors, and the age and overall health of the patient [7,8]. Therefore, a comprehensive evaluation by a fertility specialist is necessary to determine the best treatment approach for each patient.

This study aimed to assess the effectiveness of hysteroscopic surgery for intrauterine lesions on pregnancy rates in patients with primary infertility and to identify potential predictors of treatment success. A clearer understanding of these outcomes may help optimise treatment strategies and improve the chances of achieving pregnancy.

## Materials and methods

Study design and study setting

This retrospective case-control study was conducted at a specialised fertility clinic within Salmaniya Medical Complex (SMC), Bahrain's main government hospital. The Research Ethics Committee, Government Hospitals, Bahrain, issued approval (47090523). As the largest tertiary hospital in the country, SMC handles the highest volume of patients and receives referrals from all primary healthcare centres and private hospitals and clinics nationwide. The study population was identified from the operating room registry, a comprehensive record of all surgeries performed at SMC.

Study participants

This study involved a total of 113 patients of reproductive age who were experiencing primary infertility. These patients underwent hysteroscopic resection of submucous myomas, polypectomy, or resection of intrauterine septa at SMC between September 2018 and February 2023.

The electronic medical records of these patients were reviewed. The inclusion criteria focused on women of childbearing age with primary infertility attributed to intrauterine lesions (polyp, intrauterine septum, or submucous myoma). Exclusion criteria included cervical stenosis, pelvic inflammatory disease, cervical tumours, current pregnancy, uterine haemorrhage, secondary infertility, prior cervical surgery, and male factor infertility.

Data collection

Data were collected from September 2018 to September 2023 using SMC's operating room registry and electronic medical records (I-Seha). A structured data collection form was used to systematically extract relevant information, including demographic characteristics such as age, BMI, nationality, duration of infertility, type of intrauterine lesions, surgical procedures performed, pregnancy rates, and pregnancy outcomes (number of foetuses, live births, miscarriages, preterm deliveries). Additionally, the mode of delivery was recorded.

Statistical analysis

The descriptive data analyses were conducted using IBM SPSS Statistics for Windows, Version 27 (IBM Corp., Armonk, NY, USA). Demographic, health-related, and pregnancy outcomes were explored. Categorical variables, such as nationality and mode of delivery, were summarised using frequency and percentage distributions for each category.

The associations between variables were examined using the Chi-square test to identify significant differences among the groups. An alpha level of 0.05 was set as the threshold for statistical significance in all tests.

Given the sample size, multivariate regression analysis was not feasible; therefore, potential confounding variables such as age, BMI, and comorbidities were not adjusted for, which should be considered a study limitation.

## Results

The study included 113 women who underwent hysteroscopy (Tables 1, 2). Most participants were over the age of 30 (n = 82, 72.6%), and the majority were Bahraini citizens (n = 101, 89.4%). A large proportion of the women were overweight or obese, with a BMI ≥25 (n = 93, 82.3%).

**Table 1:** Socio-demographic characteristics of the participants (categorical variables) Chi-square tests of independence were used to examine associations between each variable and pregnancy outcome. Reported as χ² (df). Statistical significance was set at p < 0.05.

Variable		Total		Pregnancy rate				p-value	Chi-square (χ², df)
				Negative		Positive			
		n	%	n	%	n	%		
Age group	=<25 years	7	6.2%	0	0.0%	7	100.0%	0.099	4.66 (2)
	26-30 years	24	21.2%	8	33.3%	16	66.7%		
	>30 years	82	72.6%	33	40.2%	49	59.8%		
Nationality	Bahrain	101	89.4%	38	37.6%	63	62.4%	0.531	0.73 (1)
	Non-Bahraini	12	10.6%	3	25.0%	9	75.0%		
BMI	<25	20	17.7%	4	20.0%	16	80.0%	0.125	2.71 (1)
	=>25	93	82.3%	37	39.8%	56	60.2%		
Procedure	Diagnostic hysteroscopy + endometrial scratching	25	22.1%	12	48.0%	13	52.0%	0.175	2.78 (4)
	Myomectomy	7	6.2%	1	14.3%	6	85.7%		
	Polypectomy	40	35.4%	13	32.5%	27	67.5%		
	Polypectomy, myomectomy	10	8.8%	6	60.0%	4	40.0%		
	Septum resection	31	27.4%	9	29.0%	22	71.0%		
Duration of infertility	=<3 years	18	15.9%	9	50.0%	9	50.0%	0.370	1.98 (2)
	4-6 years	45	39.8%	14	31.1%	31	68.9%		
	>6 years	50	44.2%	18	36.0%	32	64.0%		

**Table 2:** Pregnancy rates by type of hysteroscopic procedure Chi-square test of independence; values reported as χ² (df). Statistical significance set at α = 0.05.

Procedure	Total		Pregnancy rate				p-value	Chi-square (χ², df)
			Negative		Positive			
	n	%	n	%	n	%		
Diagnostic hysteroscopy + endometrial scratching	25	22.1%	12	48.0%	13	52.0%	0.175	2.78 (4)
Myomectomy	7	6.2%	1	14.3%	6	85.7%		
Polypectomy	40	35.4%	13	32.5%	27	67.5%		
Polypectomy, myomectomy	10	8.8%	6	60.0%	4	40.0%		
Septum resection	31	27.4%	9	29.0%	22	71.0%		

The most common procedure performed was polypectomy (n = 40, 35.4%), followed by septum resection (n = 31, 27.4%). The largest subgroup based on infertility duration included those with >6 years of infertility (n = 50, 44.2%).

The youngest age group (≤25 years) had the highest post-hysteroscopy pregnancy rate (n = 7, 100%), compared to n = 16, 66.7% in the 26-30 years group and n = 49, 59.8% in the >30 years group. 

Higher pregnancy rates were observed in the lower BMI group (<25) with n = 16, 80.0%, compared to the higher BMI group (≥25) with n = 56, 60.2%. The highest pregnancy rates were seen in patients who underwent myomectomy (n = 6, 85.7%) and septum resection (n = 22, 71.0%), while pregnancy rates were also observed following polypectomy (n = 27, 67.5%), diagnostic hysteroscopy with endometrial scratching (n = 13, 52.0%), and combined polypectomy and myomectomy (n = 4, 40.0%).

Pregnancy rates by infertility duration showed the highest rate in the 4-6 years group (n = 31, 68.9%), followed by the >6 years group (n = 32, 64.0%) and the ≤3 years group (n = 9, 50.0%).

Overall, while several subgroup differences were observed (e.g., age, nationality, BMI, and infertility duration), none reached statistical significance.

Characteristics of women with primary infertility who underwent hysteroscopy (Tables 1, 2).

Positive pregnancy outcomes

A significantly higher proportion of pregnancies resulted in term deliveries (n = 45, 62.5%) compared to preterm deliveries (n = 13, 18.1%), miscarriages (n = 13, 18.1%), and ectopic pregnancies (n = 1, 1.4%), suggesting favourable gestational outcomes following hysteroscopy (p < 0.001).

Most pregnancies were conceived through IVF (n = 37, 51.4%), followed by spontaneous conception (n = 27, 37.5%), and induced pregnancy (n = 8, 11.1%), with a statistically significant distribution across conception types (p < 0.001). These findings may reflect the role of hysteroscopy in optimising conditions for successful conception, particularly in assisted reproductive techniques.

Regarding mode of delivery, lower segment caesarean section (LCS) was more frequent (n = 37, 63.7%) than normal vaginal delivery (NVD, n = 21, 36.2%) (p = 0.071). The majority of pregnancies resulted in live births (n = 58, 80.6%), a statistically significant outcome (p < 0.001). Singleton pregnancies (n = 53, 73.6%) were significantly more common than twin pregnancies (n = 19, 26.4%) (p < 0.001).

These reproductive outcomes, summarised in Table 3, indicate that hysteroscopy may contribute to improved gestational profiles and successful conception, particularly when integrated into fertility treatment protocols.

**Table 3:** Reproductive outcomes following operative hysteroscopy Chi-square tests of independence were used. Values reported as χ² (df). Statistical significance was considered at α = 0.05. *denotes statistically significant results. IVF: in vitro fertilisation; NVD: normal vaginal delivery; LCS: lower segment caesarean section

Outcomes positive pregnancies (n=72)		n	%	p-value	Chi-square (χ², df)
Outcome of pregnancy	Term	45	62.5%	<0.001*	59.3 (3)
	Preterm	13	18.1%		
	Miscarriage	13	18.1%		
	Ectopic pregnancy	1	1.4%		
Type of conception	Spontaneous	27	37.5%	<0.001*	18.1 (2)
	IVF	37	51.4%		
	Induced pregnancy	8	11.1%		
Mode of delivery	NVD	21	36.2%	0.071	4.41 (1)
	LCS	37	63.7%		
Live birth	No	14	19.4%	<0.001*	26.8 (1)
	Yes	58	80.6%		
Number of foetuses	Single	53	73.6%	<0.001*	16.1 (1)
	Twin	19	26.4%		

Procedures and pregnancy outcomes

The study examined pregnancy outcomes following various uterine procedures (Table 4). Among the procedures, polypectomy was associated with the highest rate of term pregnancies (n = 22, 47.8%) and spontaneous conceptions (n = 11, 40.7%). In contrast, septum resection was linked to the highest rate of preterm births (n = 11, 42.3%) and caesarean deliveries (n = 15, 40.5%).

**Table 4:** Pregnancy outcomes by type of hysteroscopic procedure Chi-square tests of independence were used to evaluate differences in outcomes across procedure types. Results are shown as χ² (df), with statistical significance set at p < 0.05. *denotes statistically significant values. IVF: in vitro fertilisation; NVD: normal vaginal delivery; LCS: lower segment caesarean section

Outcomes		Procedure									
		Diagnostic hysteroscopy + endometrial scratching		Myomectomy		Polypectomy		Polypectomy, myomectomy		Septum resection	
		N	%	n	%	n	%	n	%	n	%
Outcome	Term	7	15.6%	3	6.7%	22	48.9%	2	4.4%	11	24.4%
	Preterm	2	15.4%	2	15.4%	0	0.0%	2	15.4%	7	53.8%
	Miscarriage	3	23.1%	1	7.7%	5	38.5%	0	0.0%	4	30.8%
	Ectopic pregnancy	1	100.0%	0	0.0%	0	0.0%	0	0.0%	0	0.0%
	p-value	0.046*									
	Chi-square (χ², df)	21.3 (12)									
Type of conception	Spontaneous	6	22.2%	2	7.4%	11	40.7%	0	0.0%	8	29.6%
	IVF	7	18.9%	2	5.4%	14	37.8%	3	8.1%	11	29.7%
	Induced pregnancy	0	0.0%	2	25.0%	2	25.0%	1	12.5%	3	37.5%
	p-value	0.278									
	Chi-square (χ², df)	9.8 (8)									
Mode of delivery	NVD	3	14.3%	3	14.3%	12	57.1%	0	0.0%	3	14.3%
	LCS	6	16.2%	2	5.4%	10	27.0%	4	10.8%	15	40.5%
	p-value	0.024*									
	Chi-square (χ², df)	11.2 (4)									
Live birth	No	4	28.6%	1	7.1%	5	35.7%	0	0.0%	4	28.6%
	Yes	9	15.5%	5	8.6%	22	37.9%	4	6.9%	18	31.0%
	p-value	0.602									
	Chi-square (χ², df)	2.7 (4)									
Number	Single	12	22.6%	4	7.5%	22	41.5%	4	7.5%	11	20.8%
	Twin	1	5.3%	2	10.5%	5	26.3%	0	0.0%	11	57.9%
	p-value	0.017*									
	Chi-square (χ², df)	12.03 (4)									

While most pregnancies across all procedure types were singleton gestations, septum resection had the highest proportion of twin pregnancies (n = 11, 57.9%), significantly greater than that observed with other procedures.

Although pregnancy outcome categories such as term birth, miscarriage, or ectopic pregnancy did not show statistically significant differences across procedures, there were significant variations in the rate of twin pregnancies and mode of delivery.

Conception approach and pregnancy outcomes

The study examined pregnancy outcomes based on whether conception was spontaneous or non-spontaneous (IVF or induced), as shown in Table 5. The data indicate no statistically significant difference in the rate of term births between the two groups. In the spontaneous conception group, n = 18 (40.0%) delivered at term and n = 7 (15.6%) delivered preterm. In the non-spontaneous group, n = 27 (60.0%) delivered at term and n = 6 (13.3%) delivered preterm (p = 0.172).

**Table 5:** Association between conceiving approach and pregnancy outcomes Chi-square tests of independence were used. Values are reported as χ² (df). *denotes statistical significance at p < 0.05. NVD: normal vaginal delivery; LCS: lower segment caesarean section

Pregnancy outcomes		Conceiving method				p-value	Chi-square (χ², df)
		Spontaneous		Non-spontaneous			
		n	%	n	%		
Outcome	Term	18	40.0%	27	60.0%	0.172	5.5 (3)
	Preterm	7	53.8%	6	46.2%		
	Miscarriage	2	15.4%	11	84.6%		
	Ectopic pregnancy	0	0.0%	1	100.0%		
Mode of delivery	NVD	15	71.4%	6	28.6%	0.002*	10.7 (1)
	LCS	10	27.0%	27	73.0%		
Live birth	No	2	14.3%	12	85.7%	0.073	2.8 (1)
	Yes	25	43.1%	33	56.9%		
Number	Single	26	49.1%	27	50.9%	<0.001*	11.4 (1)
	Twin	1	5.3%	18	94.7%		

However, the mode of delivery differed significantly (p = 0.002). Among spontaneous conceptions, n = 15 (71.4%) delivered NVD and n = 6 (27.0%) by LCS. In contrast, among non-spontaneous conceptions, n = 6 (27.0%) were delivered vaginally and n = 16 (73.0%) by caesarean section.

While not statistically significant (p = 0.109), live birth rates were higher in the non-spontaneous conception group. In the spontaneous group, n = 18 (85.7%) had live births, compared to n = 40 (90.9%) in the non-spontaneous group. Overall, the spontaneous group contributed to n = 18 (43.1%) of live births, and the non-spontaneous group contributed n = 40 (56.9%).

With respect to fetal count, a significant difference was observed (p = 0.019). Twin pregnancies occurred predominantly in the non-spontaneous group (n = 18, 94.7%) compared to n = 1 (5.3%) in the spontaneous group. Singleton pregnancies were more common in the spontaneous conception group (n = 27, 49.1%) compared to n = 28 (50.9%) in the non-spontaneous group. This difference is likely attributable to embryo transfer practices in assisted reproduction, and therefore, no causal conclusions can be made regarding hysteroscopy and twin gestations.

Comorbidities

Hemoglobinopathies, including G6PD deficiency, beta-thalassemia, sickle cell trait (SCT), and sickle cell disease (SCD), were the most common comorbidities observed in n = 16 (14.2%) of participants. Hypertension (HTN) was present in n = 9 (8.0%) of the women, while hypothyroidism and diabetes mellitus (DM) were equally prevalent, each affecting n = 7 (6.2%). The most frequently identified gynaecological anomaly was arcuate uterus, found in n = 4 (3.5%) of participants.

Other comorbidities, including breast cancer, depression, and irritable bowel syndrome (IBS), were each present in n = 1-3 participants (<3%). Notably, more than half of the study population (n = 62, 54.9%) had no significant pre-existing medical comorbidities (Table 6).

**Table 6:** Comorbidities among study participants (n = 113) Breast Ca: breast cancer; DM: diabetes mellitus; HTN: hypertension; G6PD: glucose-6-phosphate dehydrogenase; MS: multiple sclerosis; SCT: sickle cell trait; IBS: irritable bowel syndrome; SCD: sickle cell disease

Comorbidity	Frequency (n)	%
Arcuate uterus	4	3.5%
Beta Thalassemia	1	0.9%
Breast Ca	1	0.9%
Depression	1	0.9%
DM	7	6.2%
HTN	9	8%
Endometriosis	1	0.9%
G6PD reduced activity	7	6.2%
Hypothyroidism	7	6.2%
MS	1	0.9%
SCT	5	4.4%
Hyperlipidemia	1	0.9%
IBS	1	0.9%
SCD	3	2.7%
Ulcerative colitis	2	1.8%
Nil	62	54.9%
Total	113	100.0

## Discussion

The results of this retrospective study demonstrate that hysteroscopic surgery for the removal of intrauterine lesions in patients with primary infertility is an effective strategy for improving pregnancy rates. This is consistent with the results of a systematic review encompassing randomised and control studies [9]. However, in this study, findings show that patients who underwent hysteroscopic surgery had a significantly higher rate of subsequent pregnancy, with an overall pregnancy rate of 53.8% after the procedure, which is comparable to or higher than the rates reported in other studies [5]. This suggests that hysteroscopic management of intrauterine lesions can be a valuable approach for enhancing fertility in patients with primary infertility. Our findings are in line with Donnez and Jadoul (2002), who emphasised the debated but clinically important role of submucous myomas in infertility and highlighted the benefits of myomectomy [10]. Similarly, Shokeir et al. (2004) demonstrated the significance of endometrial polyps in infertile women, supporting our observation of improved pregnancy and live birth rates following polypectomy [11]. Taken together, these findings suggest that hysteroscopic management of intrauterine lesions can be a valuable approach for enhancing fertility in patients with primary infertility.

Multiple previous studies have reported enhanced fertility outcomes following hysteroscopic removal of intrauterine pathologies such as endometrial polyps [11], submucous fibroids, and uterine septa. The underlying mechanism is restoring a more favourable intrauterine environment for embryo implantation and development.

Intrauterine lesions can distort the normal uterine anatomy and interfere with embryo implantation through several potential mechanisms. When examining the pregnancy rates by lesion type, the study found that patients with submucous fibroids had a pregnancy rate of 85.7% after hysteroscopic myomectomy. In comparison, those with endometrial polyps had a pregnancy rate of 76.5% following hysteroscopic polypectomy. Patients with an intrauterine septum experienced the lowest pregnancy rate at 71% after septum resection. These findings suggest that the type of intrauterine lesion may impact the magnitude of the fertility benefit achieved with hysteroscopic surgery.

Endometrial polyps can interfere with implantation and embryo development or physically obstruct the endometrial cavity [12]. Hysteroscopic polypectomy can significantly improve pregnancy rates in infertile women with endometrial polyps, with reported pregnancy rates ranging from 40% to 80% after the procedure [13-15]. Removing polyps restores the normal uterine anatomy and enhances the endometrial receptivity, thereby increasing the chances of successful implantation and pregnancy.

Submucous fibroids, or fibroids with a significant intracavitary component, can distort the uterine cavity and impair fertility by disrupting embryo implantation. Numerous studies have demonstrated that hysteroscopic myomectomy, the surgical removal of submucous fibroids, can improve pregnancy rates in infertile women [16,17], with reported high pregnancy rates. The resection of submucous fibroids restores the normal uterine anatomy, enhances endometrial receptivity, and improves the chances of embryo implantation.

A uterine septum is a congenital uterine anomaly characterised by a partition that divides the uterine cavity [12]. This can interfere with embryo implantation and increase the risk of miscarriage. Hysteroscopic resection of the uterine septum has been shown to significantly improve pregnancy rates in infertile women, with reported high pregnancy rates [6]. Removing the septum restores the normal uterine cavity, allowing for improved implantation and a more favourable environment for embryo development. The higher rates of preterm birth and caesarean section observed in our septum resection group may reflect the underlying complexity and clinical context of this surgical intervention

The findings of this study are consistent with previous research on the benefits of hysteroscopic surgery for improving fertility outcomes in infertile patients. Submucous fibroids, endometrial polyps, and uterine septum can all distort the uterine cavity and impair embryo implantation, reducing pregnancy rates. By removing these lesions hysteroscopically, the uterine cavity can be restored to a more normal configuration, facilitating embryo implantation and improving the chances of achieving pregnancy.

Limitations of the study include its retrospective nature, which may have resulted in incomplete or inconsistently documented medical records, and the potential for unmeasured confounding factors that influenced the decision to pursue hysteroscopic surgery or the subsequent pregnancy outcomes. Additionally, the relatively small sample size and lack of long-term follow-up data are limitations that should be acknowledged.

Despite these limitations, the findings of this retrospective study add to the growing body of evidence supporting the use of hysteroscopic surgery for the management of intrauterine lesions in infertile patients. The study's retrospective design, which allowed for the inclusion of patients treated over an extended time, is a notable strength that enhances the generalisability of the findings.

From a practical standpoint, these results suggest that hysteroscopic evaluation and treatment of the uterine cavity should be considered an integral part of the infertility workup and management. Given the high prevalence of intrauterine pathologies in infertile patients [9], routine hysteroscopic assessment may help identify and address underlying structural issues that could compromise fertility. This, in turn, may improve the chances of achieving a successful pregnancy, either naturally or through assisted reproductive technologies.

## Conclusions

Infertility remains a significant public health concern, with profound implications for the physical and psychological well-being of affected individuals.

This study examined reproductive outcomes following hysteroscopic procedures, including resection of submucosal myomas, polypectomy, and septum resection. An overall pregnancy rate of 64% was observed, with the majority resulting in singleton gestations. Live birth rates varied by pathology, with the highest observed after polypectomy. While these findings suggest that hysteroscopic surgery may be associated with improved fertility outcomes in women with intrauterine lesions, the retrospective design and limited sample size warrant cautious interpretation. Larger, controlled studies are needed to confirm these associations.
